# Impact of the Ultrasonic scalpel on the amount of drained lymph after axillary or inguinal lymphadenectomy

**DOI:** 10.1186/s12893-017-0222-1

**Published:** 2017-03-21

**Authors:** Olivier Gié, Marie-Laure Matthey-Gié, Pedro-Manuel Marques-Vidal, Nicolas Demartines, Maurice Matter

**Affiliations:** Department of Visceral Surgery, University Hospital of Lausanne, Rue du Bugnon 46, Lausanne, CHUV 1011 Switzerland

**Keywords:** Lymphatics, Radical lymph node dissection, Surgical morbidity, Harmonic scalpel

## Abstract

**Background:**

Seroma formation and lymphoedema are frequently encountered complications after radical lymph node dissection (RLND). Attempts to reduce the lymphatic morbidity include the use of Ultrasonic Scalpel. The aim of the present analysis was to assess the impact of the ultrasonic scalpel on the amount of drained lymph after lymph node dissection.

**Methods:**

Patients listed for a RLND or completion lymph node dissection (CLND) were enrolled in a prospective randomized trial to compare the impact of two surgical dissection techniques (USS versus control) on the amount of drained lymph. The lymph drained in 24 h was collected. Our primary endpoint was to compare the daily amount of drained lymph between the two groups. Secondary endpoints were the comparison of drained lymph with the BMI of the patients, the gender and the surgical site (axilla, groin).

**Results:**

Eighty patients were randomly assigned to the USS group or the Control (C) group. No difference was measured in the total amount of lymph drained (USS: 2908 ± 2453 ml vs. C: 3898 ± 5791 ml; *p*-value = 0.382). The result was also similar after adjusting for gender, age, and BMI. A significant higher amount of lymph was measured after inguinal dissection with USS compared to axillary (*p* < 0.001).

**Conclusion:**

The study suggests that the use of Harmonic scalpel did not influence the amount of lymph drained after RLND and not support the theory that USS induces oversealing of lymphatics.

**Trial registration:**

Clinical Trial NCT02476357. Registered 20 of February 2015.

## Background

Radical lymph node dissection (RLND) plays an essential role in staging and control of locoregional disease in both skin cancers and soft tissue tumours. The indication for RLND in the axilla or in the groin is the discovery of a clinical evident lymph node macrometastasis or a micrometastasis after lymph node biopsy. Important postoperative complication such as lymphocoele, lymphorrhoea and lymphoedema may occur in up to 50% of cases [1–3]. Lymphoedema has been shown to increase postoperative discomfort and can result in limb disability [4] or lead to septic complications as cellulitis and lymphangitis [5]. Such complications increase the costs of surgery and have a negative effect on the quality of life [6]. Attempts to reduce lymphatic morbidity (use of fibrin glue, vacuum dressing or modifications of surgical techniques) have been discussed in the literature without showing significant benefits [7, 8].

The use of the ultrasonic scalpel (USS) has been suggested to have a positive impact on morbidity, in trial data from breast surgery patients [3, 9]. However, subsequent trials have cast doubt on this effect [10, 11]. A recent randomized trial conducted on patients treated for melanoma, skin cancer and sarcoma showed a higher rate of lymphoedema in patients operated with USS [12]. The author’s hypothesis was that the USS ensures a more efficient sealing compared to diathermy, resulting in a higher rate of lymphostasys in the operated limb. In the same way a randomized trial focused on gastric cancer supports this hypothesis reporting a postoperative reduction of lymphorrhoea when USS was used [13].

The choice of dissecting device is not the sole determinant of the amount of drained lymph after RLND. When compared with axillary dissection, there is some evidence to suggest that radical inguinal node dissection is associated with an higher rate of drained lymph and consequently a higher rate of lymphoedema and lymphorrhoea. [14] It is logical to believe that a bigger limb may produce a greater amount of lymph compared to a smaller one, i.e., the lower limb compared to the upper limb. In the same way, studies focusing on breast cancer have suggested a direct correlation between body mass index and lymphatic specific morbidity [15]. However, this has not been confirmed in other trials [16, 12], and there is much debate regarding the impact of the BMI on lymphatic morbidity. Use of a Redon suction drain to evacuate the amount of lymph lost by unsealed lymph vessels after inguinal and axillary RLND is an effective approach to reduce lymphatic complications [17, 18]. The aim of this study is to analyze if the use of the ultrasonic scalpel (USS) can influence the amount of drained lymph after radical lymph node dissection when compared with classical dissection conducted with monopolar scalpel and ligature (control).

## Methods

This was a single-centre randomised controlled trial conducted in a tertiary academic institution between March 2009 and November 2013. Patients listed for an inguinal or axillary RLND or completion lymph node dissection (CLND) after positive sentinel lymph node biopsy (SLNB) for melanoma, skin cancer or sarcoma were invited to participate in a randomised controlled trial. Consenting patients were randomised into two treatment arms, a USS group and a control group. Details of the study design have been published previously [19]. The study protocol was accepted by the local ethics committee and registered under ClinicalTrials.gov (trial no. NCT02476357). Patients older than 18 years and listed for elective lymphadenectomy with capacity to provide informed consent were eligible for inclusion. Patients with insufficient follow up data or undergoing both iliac and inguinal RLND were excluded from the analysis. Patient presenting other causes of lymphoedema were also excluded. In the USS group, the dissection was conducted using an Ultrasonic scalpel (Harmonic Focus®, Ethicon Endo-Surgery (Europe) GmbH). In the control group the operation was performed using an electrocautery and ligature.

### Follow-up

Post-operatively, the amount of the drained lymph in 24 h was daily measured by dedicated staff during the hospitalisation. After the discharge the patient recorded on a monitoring sheet the daily amount of lymph present in the drain. The drain was subsequently removed when the lymph flow was less than 50 mL/24 h for 2 days. Some patients had occasional puncture of residual lymphocele thereafter.

### Endpoints and statistical analysis

Our primary endpoint was to compare the daily amount of drained lymph between the two groups. Secondary endpoints were the comparison of drained lymph with the BMI of the patients, the gender and the surgical site (axilla, groin).

Demographic data collected included age, gender, ASA classification, body max index (BMI), primary tumour and the number of lymph nodes harvested.

Statistical analysis was conducted using Stata version 14.0 for Windows (Stata Corp, College Station, TX, USA). Results were expressed as average ± standard deviation for continuous data and as number of patients and percentage for categorical data. Between-group comparisons were performed using chi-square for categorical data and Student’s *t*-test for continuous data. Between-group multivariate comparisons were performed using analysis of variance adjusting for gender, age (continuous), body mass index (continuous), procedure and sentinel node biopsy; results were expressed as adjusted mean ± standard error. Multivariable analysis of the amount of drained lymph between the two groups was conducted using a random-intercept, random-slope mixed model taking into account each patient’s individual evolution (i.e., a specific intercept and slope for each patient); results were expressed as coefficient and (95% confidence interval) for each parameter of the fixed model. Time to drain removal was assessed by Cox proportional hazards regression adjusting for gender, age, body mass index, procedure and sentinel node biopsy; results were expressed as hazard ratio and (95% confidence interval). Statistical significance was considered for a two-sided test *p*-value <0.05.

## Results

Seventy nine patients were randomly assigned to the USS group or the Control (C) group. Of the 80 eligible patients 1 patient randomized in the USS group was excluded due to insufficient data collection at home. The patient characteristics and the surgical details for included patients are summarized in Table 1. There was no significant difference in baseline characteristics between the two groups.

**Table 1:** Characteristics of the patients according to type of scalpel

	USS (*n* = 39)	Control (*n* = 40)	*p*-value
Women (%)	16 (41.0)	16 (40.0)	0.926
Age (years)	59.3 ± 15.2	61.9 ± 13.1	0.406
BMI (kg/m^2^)	25.6 ± 4.5	27.0 ± 5.0	0.184
BMI categories (%)			
-25.0[	18 (46.2)	12 (30.0)	0.315
[25–30[	14 (35.9)	20 (50.0)	
[30+	7 (18.0)	8 (20.0)	
Sentinel node biopsy (%)	14 (35.9)	14 (35.0)	0.934
Procedure (%)			
Axillary	27 (69.2)	31 (77.5)	0.406
Inguinal	12 (30.8)	9 (22.5)	

*BMI*, body mass index. Results are expressed as number of patients (%) or as average ± standard deviation. Between-group comparisons performed using chi-square test for categorical data and Student’s *t*-test for quantitative data

**Fig. 1:**
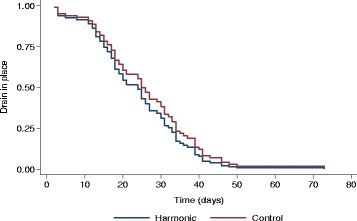
Time to drain removal according to scalpel. Comparison of control vs. harmonic scalpel using Cox proportional hazards regression adjusting for gender, age (continuous), body mass index (−25.0 [, [25–30 [and [30+), procedure and sentinel node biopsy : hazard ratio 0.85, 95% confidence interval (0.51; 1.33), *p* = 0.426

As reported in Table 2, the duration of drainage according to the type of scalpel was the same in the two groups. No differences were also seen in the average amount and in the multivariate adjusted-average amount of drained lymph between USS and control group. Analysis of variance adjusting for gender, age, BMI, procedure and previous sentinel node biopsy (Fig. 1) confirm these findings. A higher rate of drained lymph was measured after inguinal RLND with USS compared to axillary resection (Table 3).

**Table 2:** Total volume of lymph drained and duration of draining, according to type of scalpel

	Harmonic (*n* = 39)	Control (*n* = 40)	*p*-value
Total volume (ml)			
Univariate	2908 ± 2453	3898 ± 5791	0.382
Multivariate-adjusted	2864 ± 715	3941 ± 706	0.293
Duration of draining (days)			
Univariate	23 ± 14	28 ± 15	0.195
Multivariate-adjusted	24 ± 2	27 ± 2	0.290

Results are expressed as average ± standard deviation or as multivariate-adjusted average ± standard error. Between-group comparisons performed using analysis of variance adjusting for gender, age (continuous), body mass index (continuous), procedure and sentinel node biopsy

**Table 3:** Factors associated with lymph draining

	Harmonic	*p*-value	Control	*p*-value
Procedure (inguinal vs. axillary)	2285 (799; 3770)	<0.001	2272 (−1930; 6475)	0.290
Sentinel node biopsy (no vs. yes)	388 (−1126; 1903)	0.620	24 (−3470; 3517)	0.990
Gender (woman vs. man)	300 (−1165; 1764)	0.690	2132 (−1362; 5627)	0.230
Age (per year)	−9 (−56; 37)	0.700	34 (−102; 169)	0.630
BMI (per kg/m^2^)	122 (−36; 279)	0.130	−32 (−405; 342)	0.870
Day	−123 (−144; −103)	<0.001	−121 (−148; −95)	<0.001

*BMI*, body mass index. Results are expressed as coefficient and 95% confidence interval (CI) of the fixed parameters. Statistical analysis by a mixed model using individual origin and day slope as random effect parameters. Test for interaction between type of scalpel and day: z = 0.06, *p*-value = 0.953

## Discussion

Lymphoedema and seroma formation are common complications following radical lymph node dissection. They lead to prolonged hospital stay, postoperative discomfort and higher costs. Overall techniques to reduce the problem of lymphatic leak after RLND include regular needle drainage of lymphocele or leaving a suction drain into place. Reduction of the quantity and duration of lymphatic complications will benefit quality of life and costs. In order to reduce the amount of drained lymph and the duration of lymph aspiration, some authors have proposed to avoid level III dissection in the axilla [20], harvesting a lower number of lymph node with a non negligible risk of nodal recurrence [21].

There is a paucity of data regarding the potential benefit of ultrasonic scalpels in reduction of lymphatic complications. A systematic review published by Kuroi et al. in 2006 suggested that Ultrasonic devices generate lower thermal injuries compared to electrocautery and by consequence have a lower rate of lymphatic complications [22]. This hypothesis was based on a case control study conducted on 139 patient undergoing level I and II axillary dissection for breast cancer that showed a significant decrease in drainage output in patients operated with the ultrasonic scalpels [3]. Similar results were found in three other prospective studies [9, 23, 24]. Iovino et al. reported a benefit for ultrasonic devices for axillary RLND in terms of drained lymph and seroma formation and suggested that ultrasonic scalpels produce an effective sealing of lymphatic vessels, including both those directed to the apex of the axilla and those draining the lymph from the mammary gland, the fascia, and the pectoral muscles to the axillary lymph nodes. These encouraging results were not confirmed by several prospective studies and meta-analysis which compared the amount of drained lymph after mastectomy using ultrasonic devices or electrocautery. Adwani et al., and several subsequent studies report no statistically significant reduction in the drainage volume and rate of seroma development [10, 25–30]. Furthermore, a recent trial [19] has shown a higher rate of lymphoedema after lymphatic dissection conducted with ultrasonic scalpels. The higher rate of lymphoedema in the ultrasonic scalpel group was thought to be explained by an oversealing of the lymphatic vessels. Another hypothesis is that USS could influence lymphangiogenesis. Previous evidence from the literature reported a reduction of 20% of lymph volume with USS, compared to the control group [3]. Based on those results and adopting a 2-sided type I error (α) of 0.05, with a sample size of 80 patients, the calculation yielded a power of 70% for the present analysis.

## Conclusion

Conducted in a homogeneous group of patients operated systematically by the same surgeon, this study suggests that the use of Harmonic scalpel did not influence the amount of lymph drained after RLND and not support the theory that USS induces oversealing of lymphatics. A subgroup analysis comparing BMI, age, gender, previous lymph node biopsy and type of scalpel failed to show any significantly difference between the ultrasonic scalpel and the electrocautery.

## Abbreviations

CLND: Completion lymph node dissection
RLND: Radical lymph node dissection
SLNB: Sentinel lymph node biopsy
USS: Ultrasonic scalpel
